# Characterization of *Monkeypox virus* dissemination in the black-tailed prairie dog (*Cynomys ludovicianus*) through *in vivo* bioluminescent imaging

**DOI:** 10.1371/journal.pone.0222612

**Published:** 2019-09-26

**Authors:** Zachary P. Weiner, Johanna S. Salzer, Elizabeth LeMasters, James A. Ellison, Ashley V. Kondas, Clint N. Morgan, Jeffery B. Doty, Brock E. Martin, Panayampalli Subbian Satheshkumar, Victoria A. Olson, Christina L. Hutson

**Affiliations:** 1 Poxvirus and Rabies Branch, Centers for Disease Control and Prevention, Atlanta, Georgia, United states of America; 2 Laboratory Leadership Service assigned to Poxvirus and Rabies Branch, Centers for Disease Control and Prevention, Atlanta, Georgia, United states of America; University of Liverpool, UNITED KINGDOM

## Abstract

*Monkeypox virus* (MPXV) is a member of the genus *Orthopoxvirus*, endemic in Central and West Africa. This viral zoonosis was introduced into the United States in 2003 via African rodents imported for the pet trade and caused 37 human cases, all linked to exposure to MPXV-infected black-tailed prairie dogs (*Cynomys ludovicianus*). Prairie dogs have since become a useful model of MPXV disease, utilized for testing of potential medical countermeasures. In this study, we used recombinant MPXV containing the firefly luciferase gene (*luc*) and *in vivo* imaging technology to characterize MPXV pathogenesis in the black-tailed prairie dog in real time. West African (WA) MPXV could be visualized using *in vivo* imaging in the nose, lymph nodes, intestines, heart, lung, kidneys, and liver as early as day 6 post infection (p.i.). By day 9 p.i., lesions became visible on the skin and in some cases in the spleen. After day 9 p.i., luminescent signal representing MPXV replication either increased, indicating a progression to what would be a fatal infection, or decreased as infection was resolved. Use of recombinant *luc+* MPXV allowed for a greater understanding of how MPXV disseminates throughout the body in prairie dogs during the course of infection. This technology will be used to reduce the number of animals required in future pathogenesis studies as well as aid in determining the effectiveness of potential medical countermeasures.

## Introduction

*Monkeypox virus* (MPXV) is a member of the genus *Orthopoxvirus* (OPXV), and is the causative agent of the disease monkeypox (MPX). MPX is a zoonotic disease that is endemic to forested areas of Central and West African and causes sporadic outbreaks of disease in humans in several different countries [1–3]. In humans, MPXV infection is characterized by an approximate 7–14 day incubation period followed by a prodrome of fever lasting 1–3 days [4, 5]. Soon afterwards, disseminated skin lesions appear progressing from macules to papules to vesicles to pustules, which begin to scab around 4 weeks after initial infection [5–7]. Two clades of MPXV have been identified, Congo Basin (CB) and West African (WA), with CB demonstrating higher morbidity, mortality and secondary transmission rates [8–10].

MPXV became the most significant public health threat among the OPXVs after the successful eradication of smallpox for several reasons. First, the clinical presentation of MPX in humans is similar to smallpox (caused by *Variola virus*) with the exception of lymphadenopathy which is unique to MPX [5–7]. Second, unlike smallpox which was a human specific disease, MPXV is zoonotic (likely a rodent reservoir [11]) which makes it more challenging for controlling and preventing disease through vaccination and raises the possibility of spillover from its normal ecological range. Third, MPX causes a significant case fatality rate (up to 10%) [4]. Finally, the current human population as a whole is more susceptible to MPXV infection due to the cessation of routine vaccination against smallpox in the 1970s and 80s. The facts outlined above may help explain the dramatic increase in reported MPX within multiple countries in Africa [12]. The potential for global spread of MPX has been seen previously such as in the United States [13], and more recently within the United Kingdom [14] and Israel [15]. The 2003 MPXV outbreak within the United States was caused by importation of infected rodents from Africa which led to infection of black-tailed prairie dogs (*Cynomys ludovicianus*) which were then sold as pets, subsequently infecting 37 humans [13, 16, 17].

There are numerous animal models that have been developed for the study of MPXV, including a recent study looking at bioluminescence following a MPXV infection within CAST/EiJ and African dormice [18]. Investigators found that the CAST/EiJ mice had intense light signal at the intranasal site of inoculation and virus spread rapidly to lungs and abdominal organs, which had a lower virus burden. In comparison, the dormice exhibited a greater variation of virus spread (likely due to their outbred status), a slower kinetics of viral spread, less light emission in the head and chest, and more replication in abdominal organs prior to death. Both mouse models are ideal for use with BLI, due to their small size, and can be used as a lethal animal model for testing therapeutics against MPXV. However when deciding on an animal model for testing therapeutics aimed at treating humans, there are several key characteristics that should ideally be obtained; firstly the ability to obtain disease with an infectious dose similar to that causing disease in humans; as well having a disease course, morbidity and mortality similar to that seen with human disease. Previous studies with the prairie dog MPXV model have shown that it shows remarkable similarities to the progression of human monkeypox disease. Use of this animal model has allowed us to identify informative stages of monkeypox disease such as the first occurrence of viable virus shedding following intranasal inoculation starts from the oral cavity at day 6 post infection. The presence of viremia (in this case demonstrated by the presence of virus DNA within the blood starting at day 6 post infection); and finally the production of anti-orthopoxvirus antibodies begins by day 13 post infection, which generally occurs at approximately the same time as cutaneous lesion development (day 9–13 post infection) [19–21]. This similar incubation time before development of skin lesions (a key characteristic of human monkeypox), make them an ideal animal model to study MPXV pathogenesis and therapeutic testing (especially in the ability to treat animals at rash onset), unlike other animal models such as CAST/EiJ mice and African dormice, which do not develop skin lesions. Traditional methods of studying pathogenesis have several limitations such as results being overly reliant on which tissues are sampled and the sampling time points. Likewise, it is often impossible to distinguish between areas of viral accumulation versus replication by comparing PCR and viral titer results. For these reasons, it can be difficult to determine the actual viral kinetics of infection. One way to overcome these limitations was the development and refinement of *in vivo* bioluminescent imaging (BLI) using luciferase. BLI allows for tracking of viral dissemination and replication in live animals. Use of luciferase has some limitations, such as quenching of the luciferase signal by pigmented skin or dark fur, and diminishing luminescent signal with greater tissue depth. For these reasons, prairie dogs present a challenge for luminescent experiments.

A previous study examining the use of luciferase expressing MPXV within the prairie dog used a limited number of animals and only imaged live animals externally [22]. However, because of pigmentation and tissue depth it is likely that the viral kinetics within internal organs was not accounted for. Here we describe a serial sacrifice study using luciferase expressing WA MPXV to examine the viral kinetics as they occur both internally (*ex vivo*) and externally in the living animal.

## Materials and methods

### Viruses

The WA strain MPXV-USA-2003-044, isolated during the 2003 U.S. outbreak [10, 17], was used for these experiments. A recombinant MPXV strain engineered to express Firefly luciferase (*luc+* MPXV) was generated by homologous recombination through a two-step process involving the F13L gene as previously described [23]. First, the F13L gene from MPXV-USA-2003-044 was deleted by insertion of a GFP (green fluorescent protein) gene under the influence of vaccinia virus (VACV) late promoter. The MPXV-USA-2003-044 F13L deletion virus expressing GFP was screened for by small plaque production and isolated by clonal amplification. The GFP gene was then replaced by a construct containing the firefly luciferase gene controlled by synthetic early-late promoter and the F13L gene. The recombinant MPXV-Luciferase virus was screened for by production of large plaques and isolated by clonal amplification. Prior to animal inoculation, viruses were propagated in BSC-40 cells as previously described and purified by sucrose-cushion [16].

### Animal collection and care

Wild caught, male and female juvenile black-tailed prairie dogs (PDs; *Cynomys ludovicianus*) were obtained from Texas following the collection and handling guidelines of the American Society of Mammalogists [24]. At the time of this study, animals were approximately two years old and had been pre-screened by a veterinarian, determined to be in good health status, and found negative for the presence of anti-orthopoxvirus antibodies. PDs were injected subcutaneously with a sterile passive integrated transponder tag at the base of the neck for animal identification. The average starting weight for animals challenged with WT MPXV was 1.18kg (range 0.94 to 1.32kg), and the average for *luc+* MPXV infected animals was 1.10kg (range 0.89 to 1.27kg). This study was carried out in strict accordance with the recommendations in the Guide for the Care and Use of Laboratory Animals of the National Institutes of Health. The protocol was approved by the Centers for Disease Control and Prevention Institutional Animal Care and Use Committee (Protocol number 2552HUTPRAC). Animals were provided dietary enrichment in addition to PD chow. Prior to all animal procedures, animals were initially anesthetized in their individual cages with 5% isoflurane gas. Once the animal was anesthetized, it was removed from the cage and maintained under anesthesia using 1–3% isoflurane supplied by a nosecone to allow for safe sampling and imaging procedures. For euthanasia, 100mg/kg of sodium pentobarbital was administered intracardiac, while the animals were maintained in a surgical plane of anesthesia.

### Animal inoculation

Inocula were prepared by diluting virus in sterile phosphate buffered saline (PBS) for a target dose of 5x10^4^ pfu/10 μl (actual dose confirmed by back titer 5.9x10^4^ pfu/10μl of WT and 4.3x10^4^ pfu/10μl of *luc+* MPXV). Animals were infected via intranasal route (IN) with WT or *luc+* MPXV (5μl in each nostril) while anesthetized. A total of 8 PDs were infected with WT MPXV, and 12 with *luc+* MPXV.

### Infection monitoring and luminescent imaging

Prior to imaging, PDs were anesthetized and administered D luciferin (150mg/kg; Perkin Elmer) via subcutaneous injection. After allowing the D luciferin to circulate for roughly 5 minutes, PDs were transferred into a Xenogen IVIS spectrum for imaging while maintained under general anesthesia. For *ex vivo* imaging of organs, animals were injected intracardiac with D luciferin while being maintained in a surgical plane of anesthesia, a second time before euthanasia and necropsy. Images were analyzed using Living Image software (version 4.5.5, Xenogen).

### Observations and sampling

Three animals challenged with *luc+* MPXV and one challenged with WT MPXV were pre-selected for imaging/euthanasia on days 6, 9, 12, and 17. For all days post infection, individual animals were observed daily for signs of morbidity, appetence, malaise, and clinical lesions including MPXV rash. On scheduled imaging/euthanasia days, oral swabs, weights, and lesion counts were collected from all animals while under general anesthesia before subsets of animals were sacrificed by humane euthanasia. For those animals euthanized, blood was collected prior to euthanasia, and tissue samples were collected during necropsy. Strict euthanasia criteria were applied throughout the study as follows: any animal that became unresponsive to touch, lost 25% or more of its starting body weight, or accrued a total score of 10 or above on a pain scale (which assigned point values based on changes in body weight, appearance, behavior, and clinical signs) was humanely euthanized as described above.

### Necropsy and tissue specimen collection

Necropsies on animals were performed within an ABSL-3 laboratory. Samples taken during necropsy included submandibular and mesenteric lymph nodes, tongue, heart/lung pluck, spleen, stomach, kidneys, gonads, bladder, large and small intestines, lesion tissue, and blood. Tissues were selected and processed as previously reported [19, 20]. Instruments were cleaned and decontaminated with 5% MicroChem Plus and 70% Ethanol between collections of tissue types. Tissue samples were stored at -70°C until further processing.

### Sample preparation for PCR and viral growth

Tissue processing was performed within a BSL-2 laboratory using BSL-3 work practices. Tissue samples were weighed and added to a tube containing 1ml aliquots of PBS and a SPEX bead (SPEX Sample Prep). The GenoGrinder 2000 was used following manufacturer’s instruction to create a tissue homogenate. 100μl of homogenate was then removed for DNA extraction and the remaining sample saved for viral culture. DNA extraction was performed using a Qiagen DNA Tissue Kit with a BioRobot EZ-1 Workstation. Samples were incubated with lysis buffer and proteinase K at 56°C for at least 1 hour to degrade tissue and inactivate viable virus prior to DNA extraction.

### Real Time (RT) PCR

Samples were tested for presence of viral DNA using primers and probes complementary to the conserved OPXV E9L gene [25]. Each sample was run in duplicate on a 96 well plate alongside controls using an ABI Viia7 Real Time PCR machine. A standard curve of purified MPXV DNA (1ng– 10fg) was run alongside samples to allow for quantification of viral DNA within tissues. Samples were considered positive if the CT value was less than or equal to 39. This assay has a limit of detection of 50 fg for viral DNA in tissue samples.

### Virus tissue infectivity

Tissues which tested positive for presence of MXPV DNA by RT-PCR were evaluated for presence of viable virus by titrating in tissue culture. Tissue homogenates were titered in duplicate using 10-fold serial dilutions on monolayers of BSC-40 cells, and incubated at 35.5°C and 6% CO_2_ for 72 hours. After incubation, cells were stained with crystal violet and formalin to visualize plaque formation and inactivate the viable virus. Titers were expressed as plaque forming units (pfu) per gram of tissue. Due to the formalin cytotoxicity, we have previously determined that our limit of detection for this assay is ~100 plaques/ml.

## Results

### Comparison of clinical observations and viral loads between animals infected with WT and *luc+* WA MPXV

Animals infected with WT or *luc+* MPXV exhibited similar clinical signs on sacrifice days. Similar to previous studies with this outbred animal model, there was variability seen in regard to clinical signs between animals. On day 6, 1/4 animals sacrificed exhibited clinical signs (inappetence), although viable virus was detected in 2/4 animals by culturing, with the lymph node exhibiting the highest titer in both cases (Table 1). By day 9 and 10, clinical signs were evident in 3/3 *luc+* MPXV infected animals compared to 2/3 WT MPXV infected animals. Interestingly lesions were identified on 3/3 animals infected with *luc+* MPXV and only 1/3 infected with WT, however this is consistent with previous studies where animals infected with WT MPXV developed lesions on day 9 or 10 [20] and the expression of luciferase made the identification of lesions much easier (Table 1). On days 11 through 14, all *luc+* infected animals (3/3) and most WT infected animals (4/5) sacrificed exhibited clinical signs of MPX as well as had visible lesions. Viable virus was detected in all animals via culture with the highest titers found in lesions on the tongue or skin, which varied between 10^5^ and 10^9^ pfu/gram (Table 1). On the final day of the study, day 17, 2 *luc+* animals remained and both appeared to be in recovery as earlier clinical signs (mild nasal discharge) observed between days 12 and 13 had passed and animals were bright and responsive. Skin lesions remained on both animals, however upon tissue culture of necropsy samples, viable virus was not found in as many tissues compared to previous days, confirming that animals were recovering from infection (Table 1).

**Table 1:** Clinical and laboratory findings in prairie dogs intranasally challenged with WA MPXV.

Sacrifice day p.i.	Prairie dog #	WT or Luc MPXV	Max lesions observed	Maximum pain score (day recorded)	Peak viral load^1^	Viable virus detected
6	13082	luc	0	0	Submandibular Lymph Node 1.2x10^6^	Tongue, Nostril, LN, Lung, Liver, Spleen, Kidney, Gonads
6	13084	luc	0	0	Submandibular Lymph Node 5.5x10^6^	Tongue, Nostril, LN, Liver, Spleen
6	13100	wt	0	0	NA	NA
6	13107	Luc	1	0	NA	NA
9	13014	luc	5	2 (day 9)	Skin lesion 3.2x10^5^	Tongue, Nostril, LN, Lung, Heart, Gonads, Skin lesion
9	13018	luc	>10	6 (day 9)	Skin lesion 1.1x10^9^	Skin lesion, Tongue, Nostril, LN, Liver, Spleen, Kidney, Gonads
9	13025	luc	2	1 (day 9)	Tongue 8.5x10^7^	Skin lesion, Tongue, Nostril, LN, Lung, Heart, Liver
9	13125	wt	0	0	Submandibular Lymph Node 1.3x10^6^	Tongue, Kidney, Liver, Lung, LN, Spleen
9	13097	wt	0	1 (day 6)	Nostril 2.0x10^7^	Nostril, LN, Lung, Kidney, Gonads
10	13030	wt	1	10 (day 10)	Tongue 7.5x10^6^	Gonads, Heart, Kidney, Liver, Lung, Nostril, LN, Spleen, Tongue
11	13024	luc	<10	10 (day 11)	Tongue 1.2x10^7^	Tongue, Nostril, LN, Lung, Heart, Liver, Spleen, Kidney, Skin lesion, Gonads
11	13047	luc	10	10 (day 11)	Skin lesion 5.1x10^7^	Gondads, Heart, Liver, Lung, Skin lesions, Nostril, LN, Spleen, Tongue
11	13043	wt	1	10 (day 11)	Skin lesion 1.7x10^9^	Gonads, Kidney, Nostril, LN, Spleen, Tongue, Skin lesion
12	13048	luc	5	10 (day 12)	Skin lesion 1.1x10^7^	Tongue, Nostril, LN, Lung, Spleen, Gonads, Skin lesion
12	13090	wt	0	0	Tongue 9.7x10^5^	Tongue, Nostril, Ln, Lung, Gonads
12	13017	wt	<10	10 (day 12)	Tongue 1.0x10^8^	Tongue, Nostril, LN, Lung, Spleen, Kidney, Gonads, Skin lesion
12	13002	wt	>10	10 (day 12)	Skin lesion 4.0x10^8^	Nostril, LN, Lung, Kidney, Gonads, Skin lesion
14	13022	wt	9	10 (day 14)	Skin lesion 1.2x10^9^	Gonads, Tongue, Spleen, Heart, Lung, Nostril, Nostril, LN, Skin lesion
17	13016	luc	1	4 (day 12)	Skin lesion 6.9x10^5^	LN, Heart, Lung, Skin lesion,
17	13099	luc	5	4 (day 12)	Skin lesion 1.2x10^7^	Nostril, Skin lesions

^1^Tissue with the highest titer in PFU/g at time of euthanasia

### *In vivo* imaging of *luc+* WA MPXV

**Fig 1:**
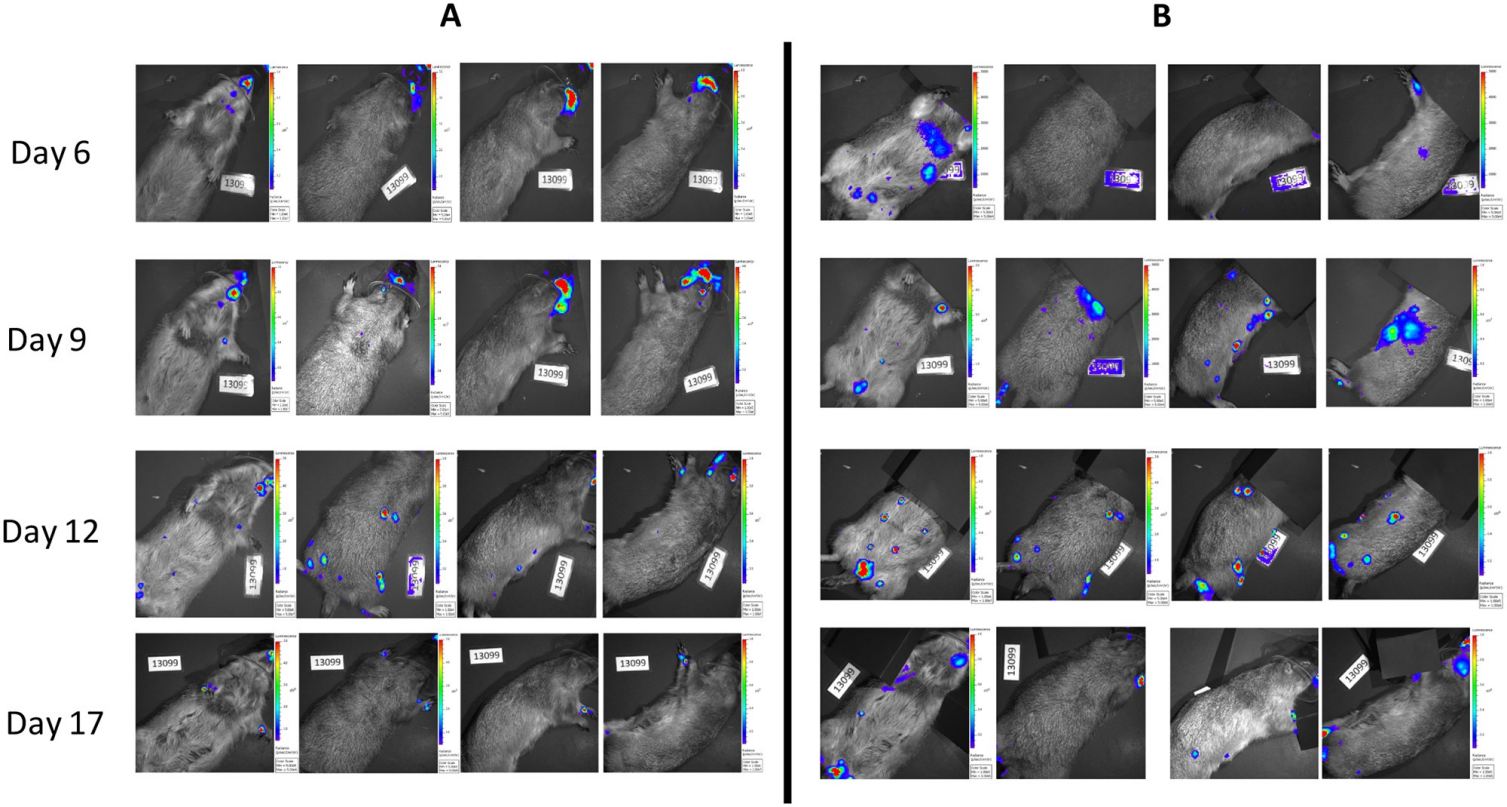
Time course of single Prairie dog infected with luc+ MPXV. Representative single Prairie dog infected with *luc+* MPXV and imaged on indicated days to determine areas of luciferase production representing areas of replicating MPXV. Images are black and white photograph of a representative prairie dog taken at each time point overlaid with a false color representation of photon emission intensity as indicated by the scale on the right in ps^-1^cm^-2^sr^-1^. Images where animals were imaged without any coverage of highly luminescent areas (A). Images where areas of high luminescence were covered by black construction paper and animals were re-imaged to visualize other areas that may require longer exposure rates (B).

**Fig 2:**
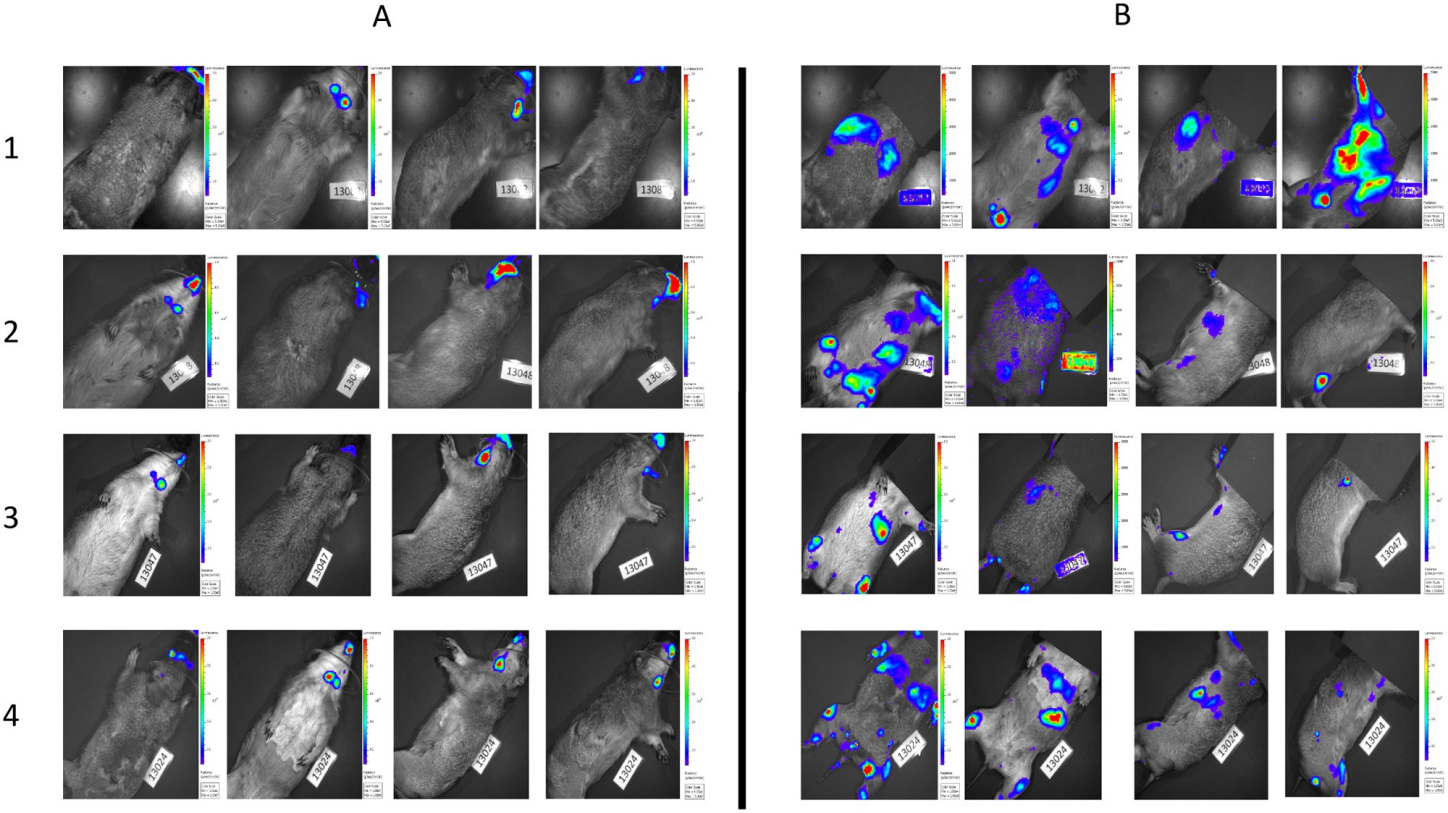
Covering initial infection site with construction paper identifies other areas of viral replication during early infection. Images are black and white photograph of a representative prairie dog taken at each time point overlaid with a false color representation of photon emission intensity as indicated by the scale on the right in ps^-1^cm^-2^sr^-1^. Individual animals 1 through 4 were imaged on day 6 post infection after injection with D luciferin. Column A represents images taken of animals with the nose area uncovered while column B shows the same animals imaged with the nose area covered by black construction paper to block the stronger signal.

**Fig 3:**
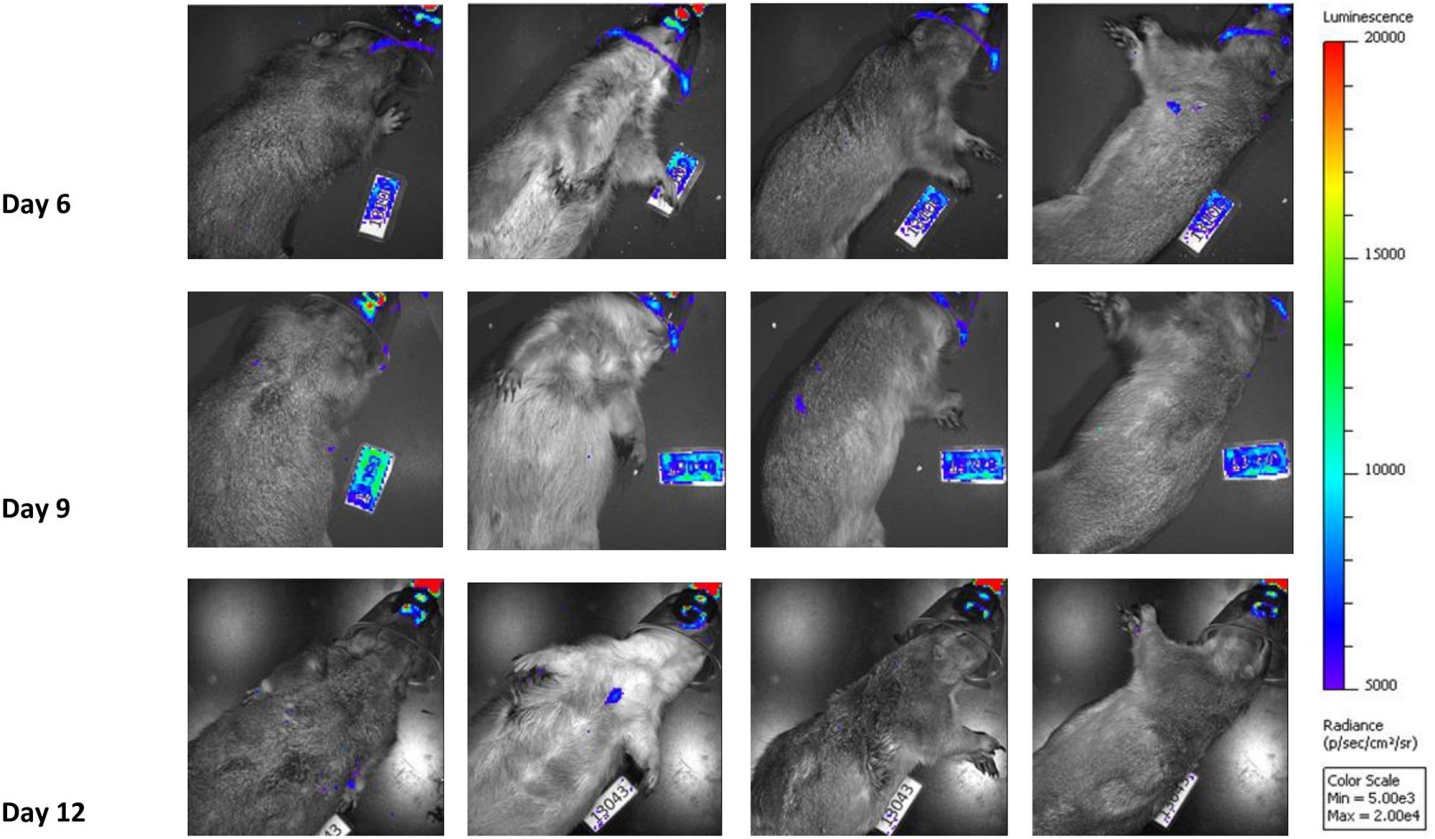
Time course of Prairie dogs infected with WT MPXV. Representative Prairie dogs infected with WT MPXV and imaged on indicated days Images are black and white photograph of a representative prairie dog taken at each time point overlaid with a false color representation of photon emission intensity as indicated by the scale on the right in ps^-1^cm^-2^sr^-1^.

Each imaging day, animals were dosed with D luciferin (WT and *luc+* MPXV infected animals), which was allowed to circulate for 5 minutes before using a Xenogen IVIS spectrum to image the animal. Each animal was imaged on the dorsal, ventral, and both lateral sides. In cases where a high luminescent signal was detected in a specific location, black construction paper was used to cover the high signal and the imaging series was repeated. This allowed for longer exposure times and areas with lower luciferase expression to be visualized which otherwise may not have been detected (Figs 1B and 2B). Similar to previously published work [22], luminescent signal was observed in all *luc+* MPXV infected animals on the first day of imaging (day 6) with a strong signal seen in the oronasal area, along with what appeared to be draining lymph nodes, then progressing to defined skin lesions between days 9 and 12, and luminescent signal decreasing by day 17 (Fig 1). At each time point, through covering high signal areas, additional areas of viral replication represented by luciferase expression were detected that were not in the original uncovered imaging series, due to these areas requiring a longer exposure time (Figs 1B and 2B). For animals imaged on days 9–12 areas of luminescence were often (but not always) associated with lesions that may have otherwise been missed during physical examination, however on days 6 and 17 these sites were often not associated with visible skin lesions (Fig 1). Animals infected with WT MPXV were imaged along with luc+ MPXV infected animals and received the same doses of D luciferin as *luc+* infected animals, however no luminescent signal over background was detected (Fig 3).

### *Ex vivo* imaging of organs identify areas of viral replication

**Fig 4:**
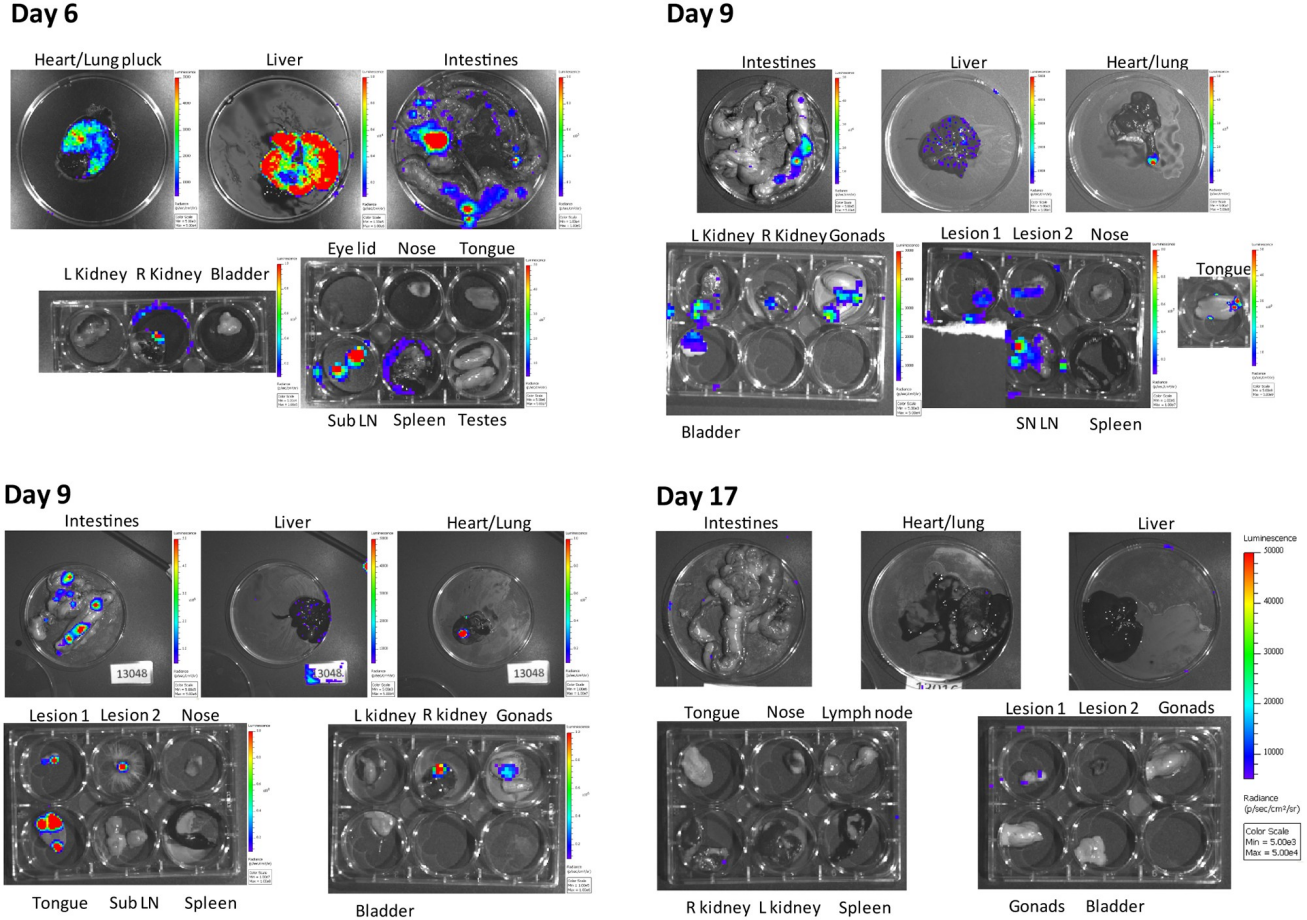
Imaging organs ex vivo reveals sites of internal viral replication. Prairie dogs infected with *luc+* MPXV were imaged on indicated days, injected via intracardiac route with additional luciferase and immediately euthanized and necropsied. Organs were removed and imaged for presence of luciferase, representing areas of replicating MPXV. Images are black and white photograph of a representative set of organs from a single prairie dog taken at each time point overlaid with a false color representation of photon emission intensity as indicated by the scale on the right in ps^-1^cm^-2^sr^-1^. In some cases organs that produced especially high levels of luminescence would be covered by black construction paper and re-imaged to visualize other organs that may require longer exposure rates.

On each imaging day 2–3 animals were selected for euthanasia/necropsy and *ex vivo* imaging of selected organs; sometimes additional animals had to be euthanized due to pain score. After *in vivo* imaging was complete, animals were given a second dose of D luciferin and immediately euthanized and necropsied. After necropsy, organs were imaged as quickly as possible (within 15 minutes after necropsy) for detection of luminescence (Fig 4). On day 6, luminescent signal was observed in all 3 animals and specifically in intestines, kidneys, heart/lung, liver, and lymph nodes. Luminescence was observed in some animals in the spleen and gonads, but was not observed in the nose and tongue of any animal (Fig 2, Table 2). Day 9 organs were similar to day 6 but with higher levels of luminescence, however luminescent signal (associated with lesions) was observed in 2/3 animals’ tongues, and 1/3 animals’ noses (Fig 4, Table 2). On days 11 and 12, luminescence was observed in every imaged organ of the animal except notably, the nose. Likewise, the spleen (1/3) and lymph nodes (2/3) had at least one animal that did not exhibit any luminescence (Fig 4, Table 2). By day 17, skin and tongue lesions along with intestines were the only areas where luminescence was observed (Table 2).

**Table 2:** Comparison of number of organs positive per time point for PCR, tissue culture and luminescence following challenge with MPXV.

	Day euthanized p.i.^1^											
	6			9			11/12			17		
	PCR Positive	TC Positive	Luciferase Positive	PCR Positive	TC Positive	Luciferase Positive	PCR Positive	TC Positive	Luciferase Positive	PCR Positive	TC Positive	Luciferase Positive
**Intestines**^2^	-	-	3/3	-	-	3/3	-	-	3/3	-	-	1/2
**Kidneys**^3^	3/3	1/3	3/3	3/3	1/3	3/3	3/3	1/3	3/3	1/2	0/2	0/2
**Heart/lung**	3/3	0/3	3/3	3/3	2/3	3/3	3/3	2/3	3/3	2/2	1/2	0/2
**Liver**	3/3	2/3	3/3	3/3	2/3	2/3	3/3	2/3	3/3	2/2	0/2	0/2
**LN**	3/3	2/3	3/3	3/3	3/3	3/3	3/3	3/3	2/3	2/2	1/2	0/2
**Nose**	3/3	2/3	0/3	3/3	3/3	1/3	3/3	3/3	0/3	1/2	1/2	0/2
**Spleen**	3/3	2/3	1/3	3/3	1/3	1/3	3/3	3/3	1/3	2/2	1/2	0/2
**Gonads**	1/3	1/3	2/3	3/3	2/3	3/3	3/3	3/3	3/3	2/2	0/2	1/2
**Tongue**	3/3	2/3	0/3	3/3	3/3	2/3	3/3	3/3	3/3	2/2	0/2	1/2

^1^ reported in number of animals positive as determined by presence of luminescence/total animals euthanized that day

^2^ no intestinal tissue was harvested for PCR analysis or viral culturing

^3^ in most cases positive signal was seen only in 1 of the two kidneys imaged

### Comparison of MPXV detection methods

After animals were euthanized and organs visualized, *ex vivo* tissues were processed for detection by RT-PCR for presence of viral DNA and tissue culture for presence of infectious virus. These results were then compared with the luminescent imaging. On animals euthanized on day 6 p.i., virtually all tissues tested positive for the presence of virus by RT-PCR, with the exception of the gonads. By tissue culture, 2/3 animals had detectible levels of infectious virus in most organs with the exception of the lungs and gonads, while in one animal levels of infectious virus were below the limit of detection in all tissues tested (Table 2). All organs tested from day 9 and days 11/12 had viral DNA detected by RT-PCR. However, several organs tested did not demonstrate presence of viable virus by tissue culture or replicating virus by luminescent signal (Table 2). Of the organs tested, the most likely to have discordant results between the three viral detection methods were the nose, spleen, and kidneys (Table 2). At each time point, these organs would be positive by RT-PCR but would be negative by tissue culture and/or luminescence. The nose was most likely to be positive by RT-PCR and tissue culture but negative for luminescence, while the kidneys were most likely to be positive by RT-PCR and luminescence but negative by tissue culture. The spleen results varied between the three methods (Table 2). By day 17 most organs were positive by RT-PCR but negative by either tissue culture or luminescence (Table 2).

### Control animals

Two animals were mock infected with PBS and sacrificed on the last day of the study (day 17). Neither of the animals demonstrated clinical symptoms of MPX at any point during the study. There was no indication of viral infection during necropsy, and no tissue samples tested positive for MPXV by RT-PCR.

## Discussion

This study expands upon previous work which used bioluminescent imaging to examine the viral kinetics of MPXV in the prairie dog model of infection [22]. Here we showed that by covering areas of high luminescence and re-imaging animals with longer exposure times additional areas of infection are captured. Likewise, including additional views (all 4 sides of animals), and *ex vivo* imaging of organs during the serial sacrifice, additional information about MPXV replication and spread within the prairie dog throughout the course of infection can be captured.

This study illustrates additional considerations when using BLI in a large animal model of infection. Particularly in the case of early infection, areas producing high levels of luminescence are capable of hiding other areas of lower levels of luminescence. In particular, on days 6 and 9 of infection viral replication occurred at a high level in the nasal regions and regional lymph nodes; these organs are at relatively shallow tissue depth and do not absorb as much light as deeper tissues. When using an auto exposure setting on the imager, these areas of high luminescence will reach saturation quickly and stop exposure. Blocking areas of high luminescence with black material allows for longer exposure times and researchers to visualize areas of low luminescence as was illustrated in Figs 1 and 2. Because of the low levels of luminescence visualized at areas distant to the nasal region on day 6 (Fig 2), and the lack of high levels of luminescence detected in corresponding organs from these distant areas *ex vivo*, it is likely the signal represents early viral infection of the lymphatic system, as has previously been suggested (12), although this will require additional study to confirm and characterize.

**Fig 5:**
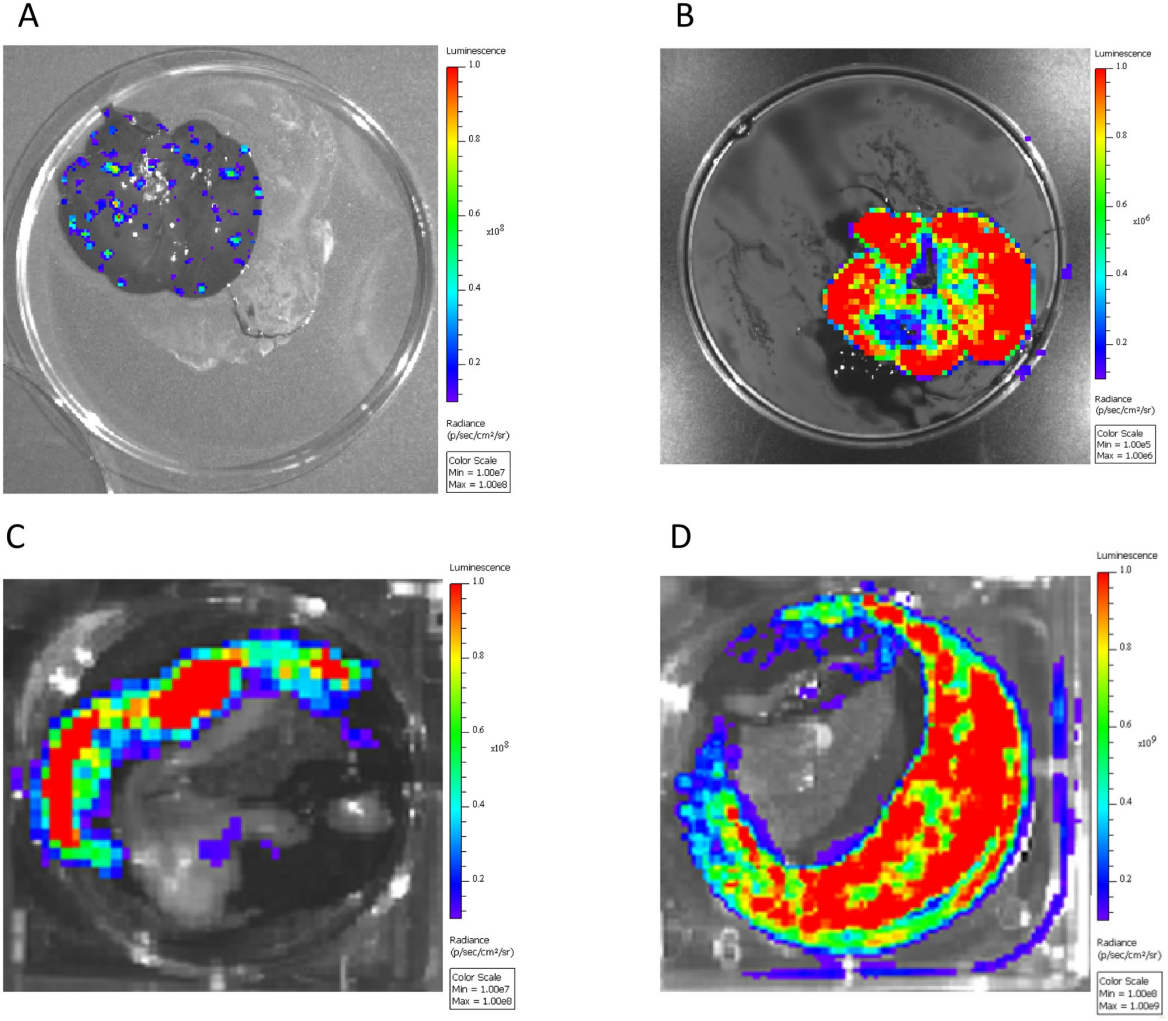
Diffuse vs uniform viral replication in organs. Prairie dogs infected with *luc+* MPXV were imaged on indicated days, injected via intracardiac route with additional luciferase and immediately euthanized and necropsied. Organs were removed and imaged for presence of luciferase, representing areas of replicating MPXV. Images are black and white photograph of a representative set of organs from a single prairie dog taken at each time point overlaid with a false color representation of photon emission intensity as indicated by the scale on the right in ps^-1^cm^-2^sr^-1^. A and C, Liver and spleen from an animals euthanized on day 9 and 11 respectively, representing diffuse multifocal MPXV replication. B and D, Liver and spleen from an animals euthanized on day 6 and 9 respectively representing uniform viral replication throughout the whole organ.

Contrary to previous investigations [22], these experiments were able to visualize luminescence in the organs of prairie dogs after necropsy. These images revealed that replication of virus can vary by organ type. Most commonly observed, viral replication was diffuse and multifocal (Fig 5A and 5C), however in rare cases (3 out of all organs imaged), it appeared to occur uniformly throughout a whole organ (Fig 5B and 5D). It is unclear if this phenomenon represents a uniform infection, or if it represents a severe multifocal infection that merged together. These data stress the importance of adequate sampling of animal tissues. Previous studies where viral DNA was detected, but virus replication was not detected by BLI and/or infectious virus was not detected by tissue culture [20, 22] may indicate areas where inactivated virus is accumulating rather than active viral multiplication. Further studies will have to determine the cause of these discordant results. Although less opportunity for sampling bias exists during BLI, it can be challenging to determine exactly where the bioluminescent signal is originating from, especially internally from larger animal models such as prairie dogs. Because of this, *ex vivo* imaging of tissues gives additional data. However, one must be careful of interpretation of this data as well. For instance, the *ex vivo* nose sample was predominately negative according to luminescence, but the necropsy method used only the very external section of the nares. We have previously seen that MPXV heavily infects the nasal-associated-lymph tissue (NALT) within this animal model following intranasal challenge [26]. Therefore, there was likely bioluminescent signal within the NALT, which was not adequately captured with our necropsy techniques used for *ex vivo* imaging the nose. This would possibly explain the intense signal seen within the nasal-oral area of the whole imaged animal.

Tissues were not uniformly positive by the three detection methods used in this study. In all cases, RT-PCR was the most sensitive detection method; some organs would have infectious virus detected by tissue culture but not BLI, or vice versa. This is most likely reflective of the relative strengths of each detection method. RT-PCR is the most sensitive method and is able to detect virus that is replicating, within an infectious virion, or possibly even virus in the process of being destroyed. Tissue culture can only detect infectious virions, but cannot determine if they are representative of sites where active replication is/were occurring versus having been shed from elsewhere. Additionally, tissue culture can be negatively impacted depending on the sample type (i.e., blood can lyse the tissue culture monolayer, thereby impacting the limit of detection in this assay). Luminescence can only be detected in areas where active viral replication is occurring, but does not necessarily indicate presence of infectious virus. This illustrates the importance of including BLI in MPXV studies, because it allows for researchers to get a better understanding of the kinetics of infection. For instance, cases in which an organ is positive by RT-PCR and tissue culture but not BLI, this would indicate areas where infectious virus particles are accumulating but no active infection is present. Organs that are RT-PCR positive, BLI positive, but culture negative may indicate areas of active viral replication that are either in early stages or the immune system is destroying virus particles as they form. Lastly, cases in which tissues are RT-PCR positive but negative by culture and BLI may indicate areas where infection has been or is in the process of being cleared. Future studies will be needed to confirm these theories. This study did not include histology of tissue samples. However, because there were no noted differences between disease progression and mortality between WT MPXV and *luc+* MPXV we would expect that the findings would be similar to a previous study by our group using prairie dogs inoculated intranasally with the same strain WA MPXV [19].

Other investigators have utilized BLI technology to investigate the potential reservoir of MPXV, which is currently unknown. In one study, investigators looked at the spread of MPXV within African rope squirrels (Funisciurus spp.) with the Congo Basin clade of MPXV (expressing the luc gene) using both intradermal and intranasal inoculation routes. MPXV infection caused mortality and moderate to severe morbidity, long periods of viral shedding and similar development of skin lesions as is seen in the prairie dog MPXV model [22]. In another study from the same investigators, Gambian pouched rats were similarly challenged with the Congo Basin clade of MPXV (expressing the luc gene) using both intradermal and intranasal inoculation routes [27]. In the Gambian pouched rats, BLI was able to identify viral replication in the skin before a grossly visible lesion had developed and also identified BLI likely in close proximity to the primary site of infection (likely in lymphatic tissue); both of which were findings in our study with the prairie dogs. These studies had numerous differences from our current study with the prairie dog, namely ours was a serial sacrifice study and we challenged with the West African MPXV clade. However as we have highlighted herein, studies utilizing BLI during MPXV infection of large animals such as Gambian pouched rats [27], African rope squirrels [28], and prairie dogs [22] would benefit from the techniques that we have highlighted within our manuscript. The use of covering areas of high luminescence so that less intense areas can be visualized, and utilization of *ex vivo* imaging of organs would have given the investigators a more complete picture of viral spread within the Gambian pouched rats and African rope squirrels and should be incorporated in future studies.

In summary, this study expands the potential for use of BLI in the prairie dog model of MPXV infection, especially in identifying early sites of infection, and the potential of testing new therapeutics in stopping viral spread from these sites. The primary purpose of this study was to establish techniques for using BLI with this animal model. Because the model has a similar disease progression and presentation as humans infected with MPXV, the prairie dog is useful for efficacy testing of MPXV therapeutics. Utilization of BLI during therapeutic testing will improve upon our previous studies in numerous ways, including the ability to use less animals and get real-time information without euthanizing the animal (seeing luminescent within the living animal). While skin pigmentation and fur do limit the ability to image internal organs of prairie dogs during infection [29–31], *ex vivo* imaging of organs can provide valuable insight as to where virus is replicating within the animal, its severity, and inform researchers which areas of the tissue should be sampled. Therefore for future *in vivo* studies, we will utilize *ex vivo* imaging which we have learned from the current study is critical for selecting samples for accurately confirming viability of virus within tissues. This study also shows the limitations of previous studies that relied on RT-PCR and tissue culture results to determine viral spread and load in various organs. With this current study, we have established specific techniques for using BLI with the prairie dog MPXV model, allowing us to strengthen the model when used during subsequent testing of anti-poxvirus therapeutics.
